# The role of Transfer RNA-Derived Small RNAs (tsRNAs) in Digestive System Tumors

**DOI:** 10.7150/jca.46055

**Published:** 2020-10-18

**Authors:** Ben-gang Wang, Li-rong Yan, Qian Xu, Xin-ping Zhong

**Affiliations:** 1 Department 1 of General Surgery, the First Hospital of China Medical University, Shenyang 110001, China; 2 Tumor Etiology and Screening Department of Cancer Institute and General Surgery, the First Affiliated Hospital of China Medical University, and Key Laboratory of Cancer Etiology and Prevention (China Medical University), Liaoning Provincial Education Department, Shenyang 110001, China

**Keywords:** tsRNAs, tRFs, tiRNAs, tRNAs, digestive system tumors, cancer

## Abstract

Transfer RNA-derived small RNA(tsRNA) is a type of non-coding tRNA undergoing cleavage by specific nucleases such as Dicer. TsRNAs comprise of tRNA-derived fragments (tRFs) and tRNA halves (tiRNAs). Based on the splicing site within the tRNA, tRFs can be classified into tRF-1, tRF-2, tRF-3, tRF-5, and i-tRF. TiRNAs can be classified into 5′-tiRNA and 3′-tiRNA. Both tRFs and tiRNAs have important roles in carcinogenesis, especially cancer of digestive system. TRFs and tiRNAs can promote cell proliferation and cell cycle progression by regulating the expression of oncogenes, combining with RNA binding proteins such as Y-box binding protein 1 (YBX1) to prevent transcription. Despite many reviews on the basic biological function of tRFs and tiRNAs, few have described their correlation with tumors especially gastrointestinal tumor. This review focused on the relationship of tRFs and tiRNAs with the biological behavior, clinicopathological characteristics, diagnosis, treatment and prognosis of digestive system tumors, and would provide novel insights for the early detection and treatment of digestive system tumors.

## Introduction

Transfer RNAs (tRNAs) have important roles in protein biosynthesis, which has a secondary cloverleaf structure containing three hairpin loops: a D loop, an anti-codon loop, and a TψC loop. TRNA may recognize codons of messenger RNA (mRNA) via its anti-codon loop and then the corresponding amino acid is transferred to the polypeptide synthesized in the ribosome^1^. Recently, in-depth analyses of new-generation data have revealed a large number of non-coding RNAs with unique function, including microRNA (miRNA), piwi-interacting RNA (piRNA), long non-coding RNA (lncRNA) and circle RNA (circRNA). Among them, transfer RNA-derived small RNAs (tsRNAs) have been paid increasing attention. TsRNAs can be found in a variety of organisms 2, 3. Numerous researches have demonstrated that tsRNAs could regulate cell proliferation, invasion, metastasis and gene expression, and thus play a crucial role in human cancer 4, 5. Aberrant regulation of tsRNAs has been reported in multiple cancers. Five tsRNAs were down-regulated in chronic lymphocytic leukemia and lung cancer, including ts-46, ts-47, ts-49, ts-53 and ts-101, while ts-4 was up-regulated in them 6. Ts-86 was down-regulated in breast cancer whereas ts-66 was up-regulated 6. Differentially expressed tsRNAs have the potential to be diagnostic markers and therapeutic targets of cancer 7, 8.

## Basic biological functions

### Classification of tRFs and tiRNAs

**Figure 1:**
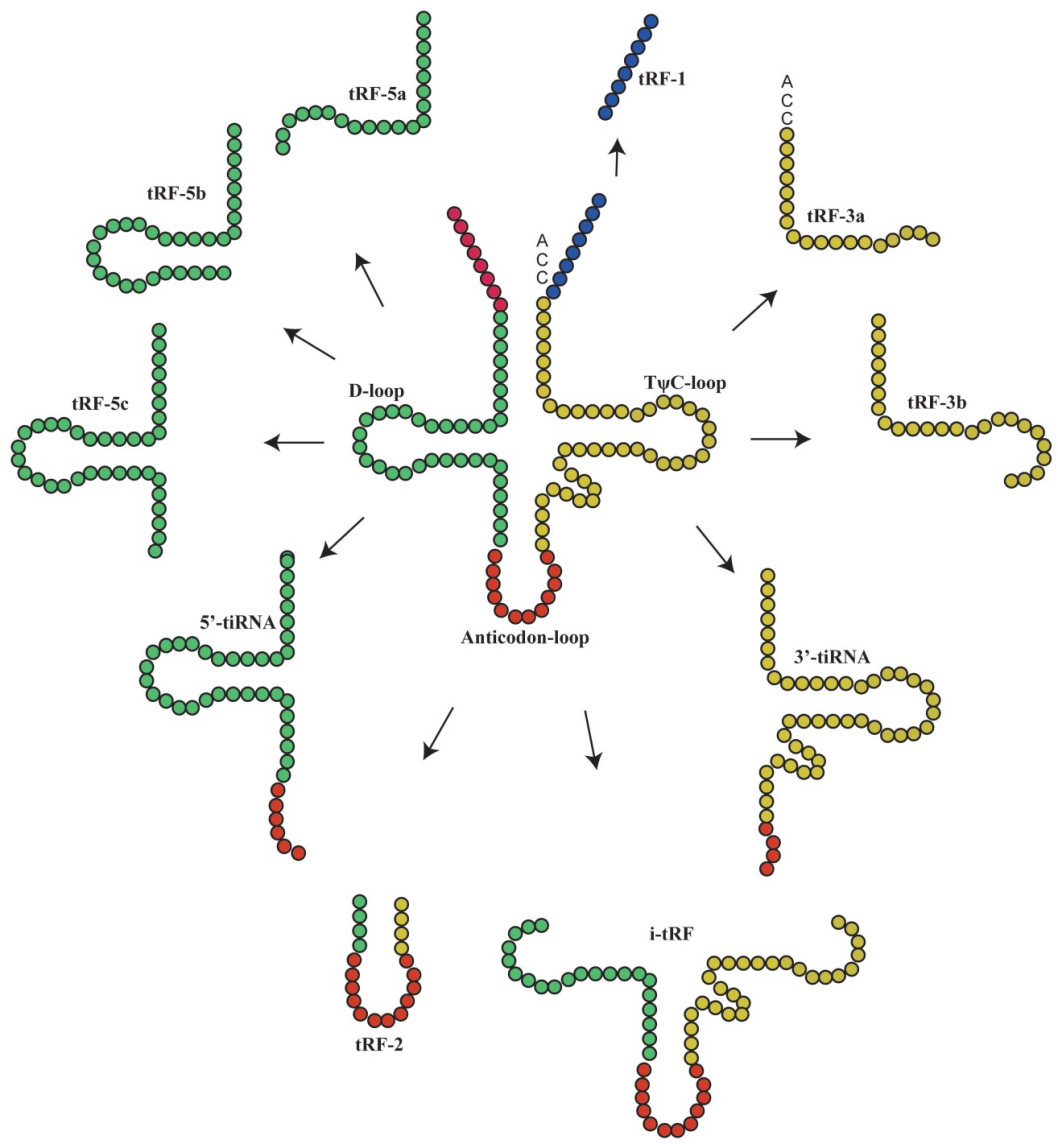
The main classification of tRNA-derived fragments (tRFs) and tRNA halves(tiRNAs). In this figure, the tRFs or tiRNAs were shown the source from tRNAs, according to the color which is the same with tRNAs'.

Under special conditions such as hypoxia, stress or UV radiation, tRNA is cleaved into small tsRNA fragments by specific nucleases^9^, which can be classified into tRFs and tiRNAs^10^, ^11^. To date, more detailed studies have been performed on tRFs than tiRNAs, as evidenced by the establishment of tRF databases^12^. TRFs are non-coding RNAs with 14-30 nucleotides in length. They are derived from the end of pre-tRNAs or mature tRNAs and can be classified into different types based on their original site in these molecules: tRF-1, tRF-3, tRF-5^13^, i-tRFs and tRF-2^14^, ^15^. TRF-3 (13-22Nucleotide[nt]) can be further classified into tRF-3 and tRF-3b, while t-RF-5 can be classified into three subtypes: tRF-5a (14-16nt), tRF-5b (22-24nt) and tRF-5c (28-30nt)^16^. TiRNAs are non-coding small RNAs with 31-40 nucleotides in length, which can be classified into 3'-tiRNAs and 5'-tiRNAs^11^, ^17^-^21^. The type of tsRNAs was shown in Figure 1.

**Figure 2:**
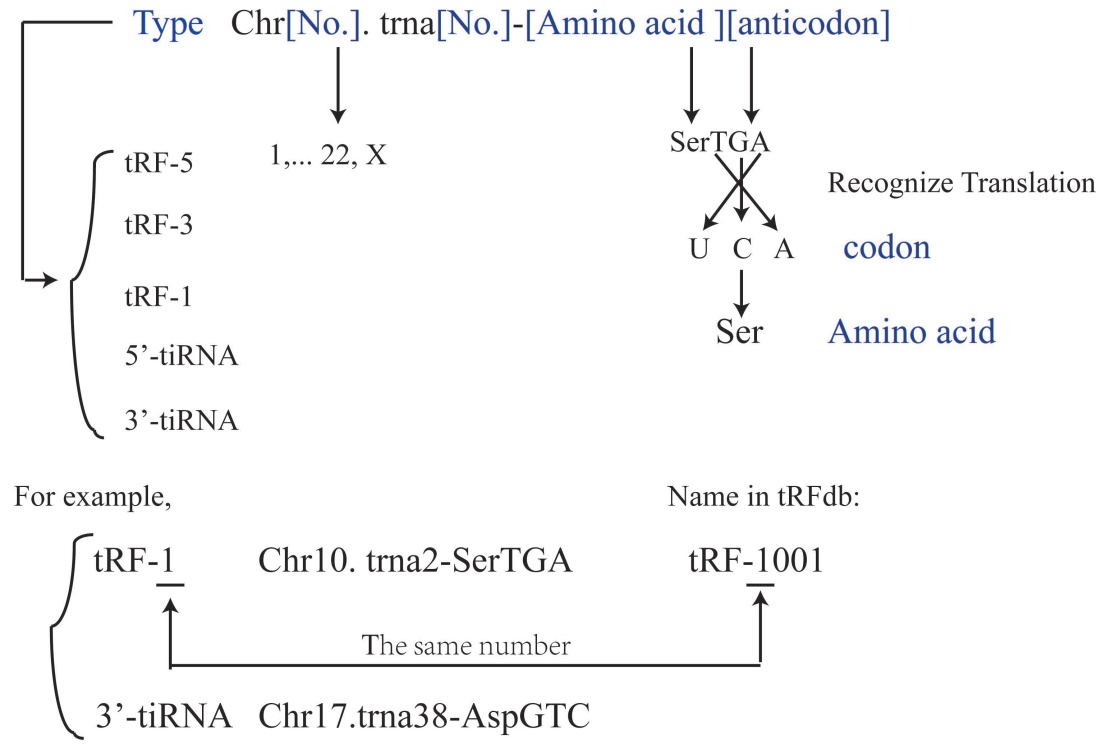
The naming rules of tRFs and tiRNAs. The name described the tRFs or tiRNAs should contain the type (tRF-5, tRF-3, tRF-1, 5'-tiRNA, 3'-tiRNA), Chromosome number, tRNA number, the amino acid carried and the anticodon carried. The anticodon is the codon which tRNA carried, then this anticodon will be translated into the codon which is matched amino acid. And every tRFs has a name in tRFdb (tRFdatabase), for example, tRF-1001 is Chr10. Trna2-SerTGA and it belongs to tRF-1 type, and the number underline in tRFdb means the type (tRF-5, tRF-3 or tRF-1).

It has been suggested that each tRF and tiRNA are produced by different enzymes. TRF-5 and tRF-3 are produced by endonucleolytic and exonucleolytic digestion of mature tRNAs, respectively. TRF-5 is mainly produced by Dicer cleavage of tRNA D loop or the stem loop between D loop and anti-codon^4^, ^11^, ^17^, ^22^-^25^; tRF-3 is produced through TψC loop cleavage by Dicer and Angiogenin (ANG), a member of ribonuclease A superfamily^4^, ^11^, ^17^, ^26^-^29^. TRF-1 is derived from the 3'-end of pre-tRNA mainly via digestion by endonuclease Z (RNase Z/ELAC2)^11^, ^17^-^21^. TRF-2 is derived from the cleavage of tRNA anti-codon^11^, ^17^, ^21^. The i-tRFs are mainly derived from the internal region (between D loop and T loop) of mature tRNA, which is across the anti-codon region^6^, ^11^, ^21^. TiRNAs are derived from mature tRNA anti-codons through digestion by ANG. The 3'-tiRNAs are generated from the 3'-end of anti-codon, while 5'-tiRNAs are from the 5'-end of anti-codon^11^, ^17^, ^21^, ^30^. The naming rules of tsRNA are listed as Figure 2 and Table 1.

### Differences and correlation between tRFs and tiRNAs

Under specific conditions, tRFs and tiRNAs are similar but not identical in terms of their structure and function. First, both of them are derived from tRNA. However, due to different enzymes and cleavage sites involved, the products differ in terms of their length and structure^17^. Second, tRFs and tiRNAs are mostly located within cells except for a small part in peripheral circulation; however, their intracellular original sites and distribution are different. TRF-5s are produced in cytoplasm and transported to nucleus via a similar mechanism for tRNA transportation^10^, ^17^, ^30^. TRF-1 might be generated in cytoplasm or nucleus. Some scholars reported that tRF-1s were generated in cytoplasm^4^. However, others believed that tRF-1s was likely to be produced in nucleus and transported to cytoplasm via specific mechanism^17^. TRF-3s are mainly distributed in cytoplasm 10, 17. TiRNAs are mainly located in cytoplasm with a small part in nucleus and mitochondria, while tiny levels are found in circulatory system^10^. However, whether the location of tRFs and tiRNAs is associated with their diverse function remains unknown. Third, both tRFs and tiRNAs have important roles in cellular transcription, gene expression and viral infection^11^, ^31^, with various mechanisms. For instance, regarding transcriptional regulation, tRF-5s were suggested to mainly inhibit transcriptional elongation, whereas 5'-tiRNAs mainly inhibits transcriptional initiation^6^, ^32^, ^33^. TRFs and tiRNA have many features in common, though further investigations are required to explore the correlation between their characteristics and disease development, especially tumor development and progression.

### Function of tRFs and tiRNAs

Although the function of most tRFs and tiRNAs have not been elucidated, accumulating evidence has suggested that they are involved in regulating cell proliferation and apoptosis, DNA damage response, gene expression and post-transcriptional modifications. TRFs and tiRNAs have the following function (Table 2).

#### Regulating cellular proliferation and apoptosis

TRFs were found to might inhibit cellular proliferation. TRF-3027 (tRNA^Gly-GCC^) may bind to AGO, an important component of RNA-induced silencing complexes (RISCs), block replication protein A1 (RPA1) to inhibit cellular proliferation, and regulate DNA damage response^6^, ^9^, ^11^, ^17^, ^21^, ^30^.

**Figure 3:**
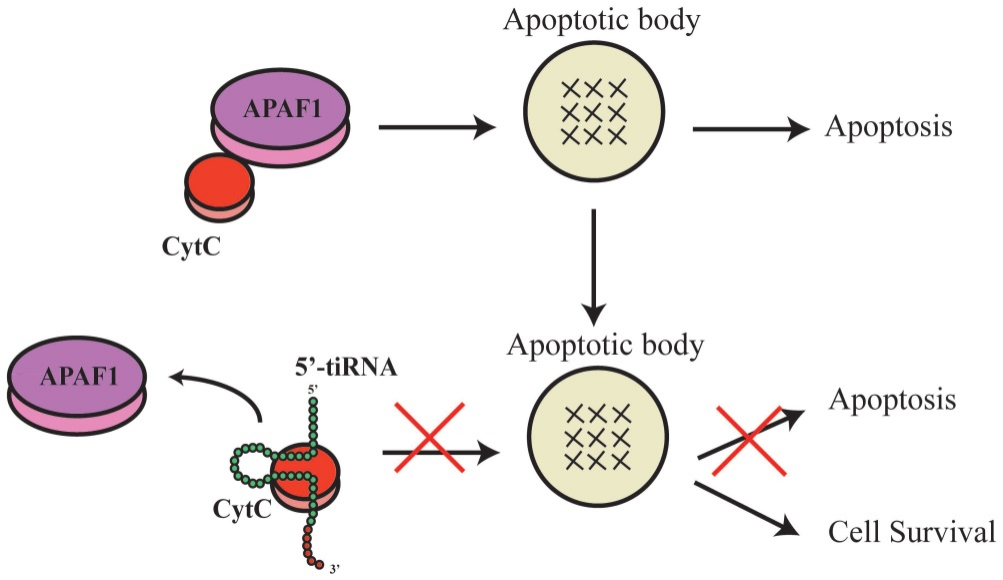
5'-tiRNA could promote the cell survival.ThetsRNAs that were derived from the tRNA also binds to the cytochrome C, which then would bind to the apoptotic protease activating factor 1 (APAF1) to form the apoptotic bodies. And the 5'tiRNA and 3'tiRNA could bind to the cytochrome C to form the ribonucleoprotein complex, inhibit formation and activity of the apoptotic bodies, and stimulate the cellular survival. APAF1, apoptotic protease activating factor 1.

TsRNAs were also indicated to induce or inhibit apoptosis. During apoptosis induction, inhibition of a specific tsRNA, 3'-tsRNA-LeuCAG, has been shown to induce apoptosis in rapidly dividing cells *in vitro* and a patient-derived orthotopic hepatocellular carcinoma model of mice. The tsRNA binds to at least two ribosomal protein mRNAs (*RPS28* and *RPS15*) to enhance their translation. A decrease in the translation of* RPS28* mRNA blocks pre-18S ribosomal RNA processing, resulting in a reduction in the number of 40S ribosomal subunits to induce apoptosis^34^. During apoptosis inhibition, tsRNAs and tRNAs have similar roles. Early studies of tRNA showed that mature tRNA bind to cytochrome C to inhibit the formation of apoptotic bodies and cysteine-aspartic protease (caspase-9) activity to stimulate cellular survival^35^. Recent studies showed that tsRNAs derived from tRNA also bind to cytochrome C^36^, which then bind to apoptotic protease activating factor 1 (APAF1) and form apoptotic bodies^36^. In response to hyperosmotic stress, ANG mediates the competitive binding of 5'-tiRNA and 3'-tiRNA to cytochrome C generating the ribonucleoprotein complex, inhibits the formation and activity of apoptotic bodies, and stimulates cellular survival^10^, ^36^-^38^ (Figure 3). However, the complete biological mechanisms remain unclear and require further investigation.

#### Function similar to miRNA and piRNA

Certain tRFs may act as miRNAs or piRNAs to bind to AGO protein, which is an important RISC^34^ with biological function. TRF-1s tend to interact with AGO3 and AGO4^6^, ^39^. TRF-5s and tRF-3s interact with AGO1, AGO3 and AGO4^17^, ^31^, ^39^. Like miRNAs, they have seed sequences complementary to target mRNAs and can be recruited to AGO complexes to regulate target RNA expression and function^17^. TRF-5 and tRF-3 both bind to miRNA via the seed region^16^, ^40^, which has been confirmed in many other tRFs. For instance, ts-53 and ts-101 not only bind to AGO proteins but also PIWIL2 (a molecule similar to piRNA), regulating target gene expression and post-transcriptional modification^6^, ^30^. However, interactions between tiRNA and AGO remain unknown and need further investigation.

#### Translation regulation

**Figure 4:**
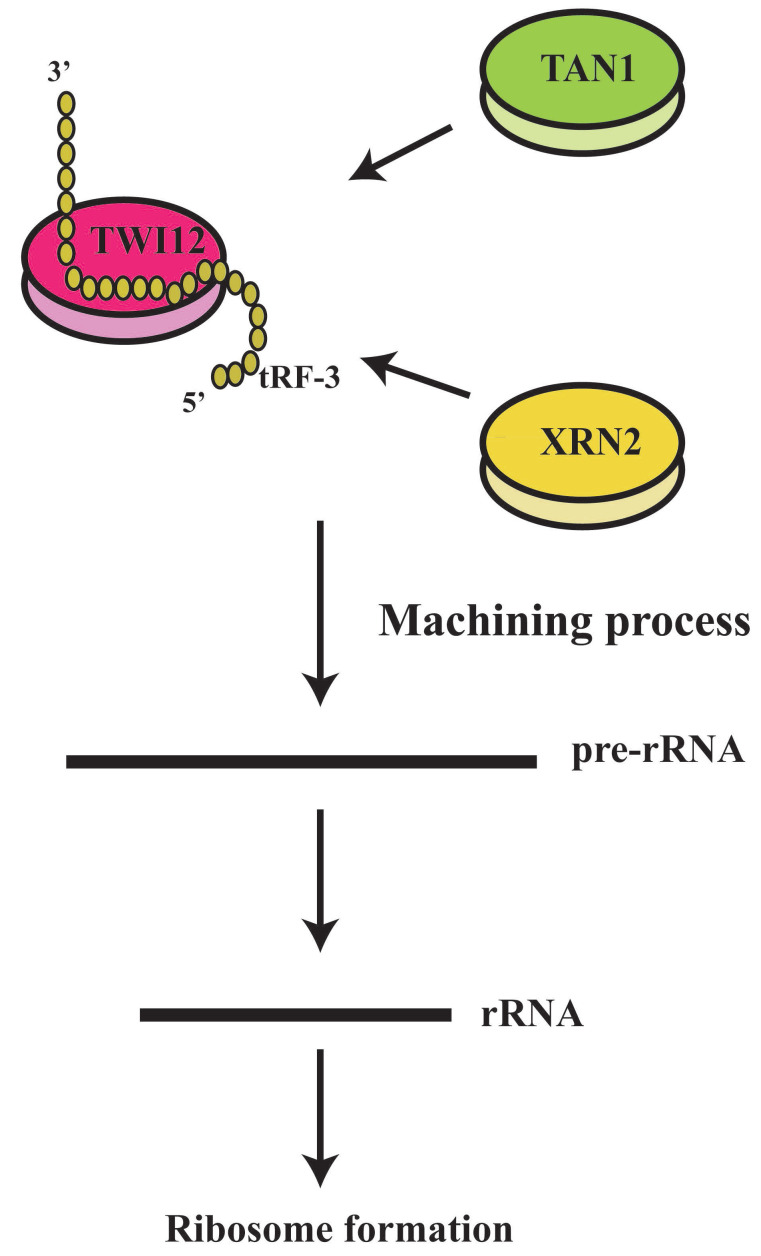
tRF-3 could regulate ribosomal functions. The tRF-3s may specifically bind to TWI12 protein (member of the AGO/PiWi protein family), and recruit Tan1 protein and exoribonuclease XRN2, to form pre-ribosomal RNA splicing complex (TXT), process pre-rRNA during rRNA synthesis, and then regulate translation.

**Figure 5:**
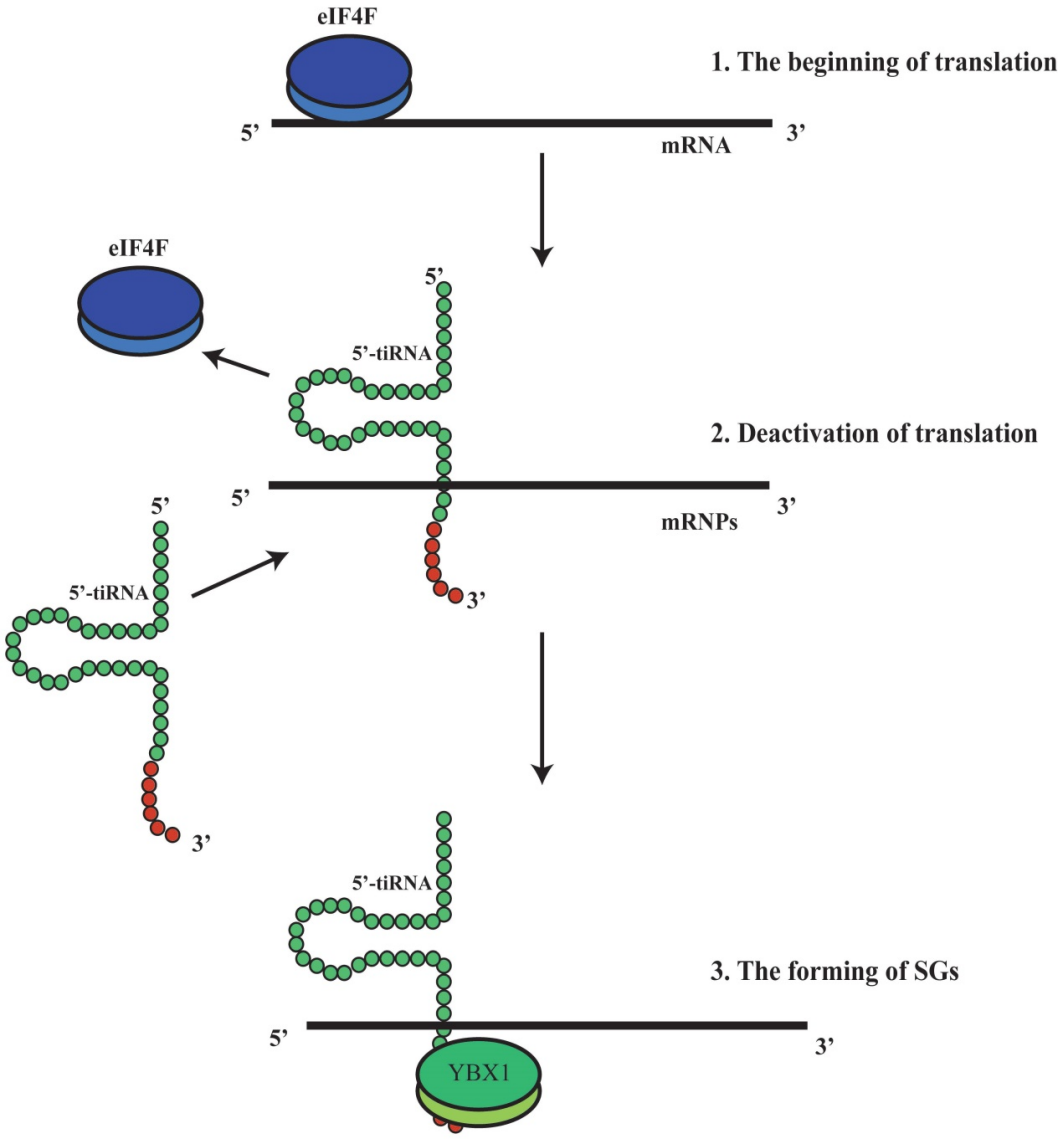
5'-tiRNA could promotethe formation of stress granules (SGs). 5'-tiRNA may replace eIF4F, which is an eukaryotic translation initiation factor, at the mRNA ^m7^GTP position, to inhibit translation initiation and produce multiple mRNA protein complexes (mRNPs).Those tiRNA may further bind to the cold shock domain (CSD) of the YBX-1 protein (RNA binding protein) to form 5'-TOG-tiRNA-protein complex and then stimulate production of the stress granules (SGs). SGs, stress granules; YBX-1, Y-Box Binding Protein 1; mRNPs, mRNA protein complexes.

It has been shown that tRFs can regulate ribosomal function. The tRF-3s may specifically bind to TWI12, a member of AGO/PIWI protein family, and recruit TAN1 protein and exoribonuclease XRN2 to form pre-ribosomal RNA splicing complex (TXT), process pre-rRNA during rRNA synthesis, and then regulate translation^41^. TRF-3 (e.g. 3'-tRNA-LeuCAG) may bind to two ribosomal protein mRNAs (*RPS28* and *RPS15*) to stimulate translation, ribosomal synthesis and cellular proliferation^10^ (Figure 4). However, tiRNAs have not been reported to regulate ribosomal synthesis^10^, ^11^, ^17^, ^41^. Some tRFs and tiRNAs may directly regulate cellular translation^33^, ^42^-^44^. Recent studies have demonstrated that 5'-tiRNAs (5'-tiRNA-Ala and 5'-tiRNA-Cys) contain 5'-terminal oligoguanine motifs (5'-TOGs) and may replace eIF4F, a eukaryotic translation initiation factor, at mRNA m^7^GTP position to inhibit translation initiation and produce multiple mRNA protein complexes (mRNPs)^45^. These tiRNAs may further bind to the cold shock domain (CSD) of YBX-1 RNA binding protein to form 5'-TOG-tiRNA-protein complexes and then stimulate the production of stress granules (SGs)^6^, ^31^, ^37^, ^38^, ^45^, ^46^ (Figure 5). 5'-tiRNAs may induce SGs assembling via phosphorylated eiF2α^6^, ^10^, ^11^, ^17^, ^38^, ^39^.

Another interesting phenomenon was found that the functional pattern was distinct from that of canonical miRNA. TRF5-GluCTC, a type of 5'-tRFs, plays a gene-silencing role on the target mRNA with complementary target sequence. Regulation studies showed the 5'-portion of miRNAs is the key determinant in target recognition, while this suggested the 3'-portion of tRF5-GluCTC is critical for its gene silencing function through a trans-silencing mechanism 3.

Compared with miRNAs and piRNAs, tRFs and tiRNAs may directly regulate cellular translation, suggesting that these small RNA molecules have more complicated regulatory potentials to maintain various biological function and thus more important roles.

## Roles in digestive tract (DT) tumors

### Roles of tiRNAs and tRFs in the cellular biology of DT tumor

TRFs and tiRNAs have important roles in tumor development and progression. However, the study on the correlation between tRFs/tiRNAs and tumor remains at an early stage. The expression levels of tRFs and tiRNAs were suggested to be abnormal in many tumor cells^47^, ^48^ and correlated with tumor cell proliferation, migration and transformation^6^, ^48^. Recent studies on DT tumor (rectal cancer) showed that tRF/*miR-1280* expression levels were decreased^5^. Furthermore, tRF/*miR-1280* inhibits the 3' UTR of *JAG2*, reducing JAG2 biosynthesis to inhibit the Notch pathway and directly inhibit proliferation, migration and epithelial-mesenchymal transformation (EMT) of rectal cancer cells^5^, ^49^, ^50^. TRF/*miR-1280* also decreases the expression of CD133+stem cell marker in CRC cells, reduces their activity and migration, and prevents formation of the microenvironment of rectal cancer cell metastasis^5^. Moreover, Ts-53 and ts-101 might have important roles in the early transformation stage of rectal cancer^48^. Ts-40 might be oncogenic during rectal cancer development^48^. Ts-36, correlated with cellular proliferation, may exert roles in the final stage of metastatic transformation of rectal cancer^48^. A research applying tiRNA sequencing was performed in 30 HCC patients (including 10 with HBV and 20 with HCV) and 9 controls, and tiRNAs were found to be the most abundant small RNAs in chronically infected liver and the abundance of 5'- tiRNAs was reduced in liver cancer. In addition, in hepatitis B-associated HCC, 5'- tiRNAs abundance was correlated with the expression of tRNA-cleaving ribonuclease, angiogenin 51. Therefore, tRFs and tiRNAs are critical for the biological behavior of DT tumor cells. In-depth investigations would benefit understanding how to exploit these molecules to interfere with tumor progression and how to select molecular targets for therapy.

### Correlation between tiRNAs/tRFs and the clinicopathological characteristics of DT tumor

In clinical case, distant metastasis of DT tumor is a major obstacle to optimal patient prognosis^52^. Thus, researchers have been exploring the correlation of tiRNAs/tRFs with the cellular invasion and distant metastasis of DT tumor. Recent studies showed that colorectal cancer had elevated ANG levels, increased the levels of 5'-tiRNA-Val, 5'-tiRNA-Cys and 5'-tiRNA-Ala, then promoted cancer cell invasion and migration without affecting cellular proliferation^52^. Additionally, 5'-tiRNA-Val levels are positively correlated with lymph node metastasis and distant metastasis of colorectal cancer cells^52^. However, the mechanism remains unclear. Further investigations would benefit the early detection of distant tumor metastasis, providing new ideas for the treatment of metastatic tumors and improving prognosis.

### Correlation between tiRNAs/tRFs and DT tumor diagnosis

Seminal exosomes were found to contain more tRFs than miRNAs, suggesting that tRFs could be stably expressed and were abundant in body fluids^53^. That makes them to be potential markers for early tumor diagnosis. Recent studies showed that 5'tRF-GluCTC and 5'tRF-ValCAC levels were decreased in colorectal cancer cells, indicating that they were involved in the development and progression of colorectal cancer and could serve as potential diagnostic markers^2^. Moreover, tiRNA-5034-GluTTC-2 was significantly decreased in gastric cancer (GC) tissue, neoplasm and GC cells, and low levels of tiRNA-5034-GluTTC-2 in GC tissue were correlated with tumor size^54^. TiRNA-5034-GluTTC-2 in GC tissue had higher sensitivity and specificity with the large area under receiver operating characteristics (ROC) curve as a tumor marker. All these findings were statistically significant, suggesting its potential as a marker of early GC^54^. Recently, it has been shown that the expression of tsRNAs in plasma exosomes of liver cancer patients is significantly higher than that in healthy controls 55. Four tsRNAs were up-regulated including tRNA-ValTAC-3, tRNA-GlyTCC-5, tRNA-ValAAC-5 and tRNA-GluCTC-5, suggesting that these differentially expressed tsRNAs have the potential to be novel diagnostic markers of liver cancer 55. Exploration on the correlation between tRFs/tiRNAs and DT cancer development would benefit identifying markers for the early diagnosis of DT cancer and optimize detection.

### Correlation of tiRNAs/tRFs with DT tumor treatment and prognosis

Investigating the role of tiRNAs/tRFs in tumor progression would improve treatment protocols and disease prognosis. Previous reports demonstrated that elevated ANG in rectal cancer increased 5'-tiRNA-Val levels, and thus established an ANG-tiRNAs-cell invasion/metastasis regulatory axis^52^. Such findings provided insights on the mechanism of rectal cancer development and revealed new therapeutic targets for colorectal cancer. TRF/*miR-1280* could inhibit the Notch pathway and subsequent EMT and distant metastasis of colorectal cancer^5^, suggesting that it could affect cancer prognosis. Quantitative PCR (qPCR) illustrated that tRFs and tiRNAs (tRF-3-LeuAAG-1-1[AS-tDR-000064], tRF-3-GlnCTG-1-1[AS-tDR-000069], tRF-3-AlaCGC-1-1[AS-tDR-000102] and tiRNA-5-ProCGG-1-1[AS-tDR-001391]) had abnormally elevated expression in cancerous pancreatic cells, but not normal pancreatic cells^56^. KEGG and GO pathway analyses showed that all the four tRFs and tiRNAs were significantly enriched in cancer-related pathways (including RAS signaling pathway, cancer pathways, axon guidance and PI3K/AKT signaling pathway). Therefore, they might be able to serve as markers for early pancreatic cancer diagnosis and provide clues for pancreatic cancer development^56^. Furthermore, the survival rate of patients with low expression of tiRNA-5034-GluTTC-2 was much higher than that of individuals with higher expression levels, indicating that tiRNA-5034-GluTTC-2 could be a potential prognostic marker of GC^54^. All the findings suggested that tRFs and tiRNAs were crucial in DT cancer treatment and prognosis.

## Summary and future directions

Several limitations should be acknowledged. First, although the sequencing has identified many tRFs and tiRNAs, bioinformatic methodology needs to be improved for more identification. Second, current study only focused on the basic biological function of tRFs and tiRNAs without the correlations with diseases, especially DT tumor. Third, the naming roles of molecules remain not unified. Fourth, we urgently need a tiRNA database and a database to integrate the tRFs (tRFdb) and tiRNAs.

In summary, tRFs and tiRNAs have important roles in gene expression, gene translation, and the development and progression of various DT tumors^17^. They affect tumor cell proliferation, migration and invasion^48^, ^57^. To date, the origin and function of tRFs and tiRNAs remain to be fully elucidated, and their roles in DT tumor progression also require further investigation. In-depth studies would benefit early detection, prognosis evaluation and DT tumor therapy, further improving prognosis and survival.

## Figures and Tables

**Table 1:** Data from the tRFdb*

tRF ID	Organism	Type	tRNA Gene Co-ordinates	tRNA Name
1001	human	tRF-1	chr10-69524261-69524342	chr10.tRNA2-SerTGA
1003	human	tRF-1	chr17-8090184-8090265	chr17.tRNA7-SerGCT
1004	human	tRF-1	chr12-98897281-98897352	chr12.tRNA5-AspGTC
				
1042	human	tRF-1	chr19-33667963-33668036	chr19.tRNA4-ThrAGT
				
3001a	human	tRF-3	chr17-8023713-8023632	chr17.tRNA42-LeuTAG
3001a	human	tRF-3	chr16-22207113-22207032	chr16.tRNA27-LeuTAG
3001a	human	tRF-3	chr5-180528840-180528921	chr5.tRNA3-LeuAAG
3001a	human	tRF-3	chr5-180524555-180524474	chr5.tRNA19-LeuAAG
3001a	human	tRF-3	chr5-180601125-180601044	chr5.tRNA16-LeuAAG
3001a	human	tRF-3	chr6-28911480-28911399	chr6.tRNA98-LeuAAG
3001a	human	tRF-3	chr5-180614701-180614782	chr5.tRNA7-LeuAAG
3001a	human	tRF-3	chr16-22308461-22308542	chr16.tRNA16-LeuAAG
3001a	human	tRF-3	chr14-21078291-21078372	chr14.tRNA1-LeuAAG
3001a	human	tRF-3	chr6-28956779-28956860	chr6.tRNA78-LeuAAG
3001a	human	tRF-3	chr6-28446481-28446400	chr6.tRNA126-LeuAAG
				
3033a	human	tRF-3	chr1-16847153-16847080	chr1.tRNA136-AsnGTT
				
5001a	mouse	tRF-5	chr13-23609561-23609632	chr13.tRNA111-MetCAT
5001a	mouse	tRF-5	chr15-69227124-69227053	chr15.tRNA876-MetCAT
5001a	mouse	tRF-5	chr13-21998980-21999051	chr13.tRNA82-MetCAT
5001a	human	tRF-5	chr8-67026223-67026311	chr8.tRNA5-TyrGTA
5001a	human	tRF-5	chr8-67025602-67025694	chr8.tRNA4-TyrGTA
5001a	human	tRF-5	chr2-27273650-27273738	chr2.tRNA2-TyrGTA
5001a	human	tRF-5	chr6-26595102-26595190	chr6.tRNA17-TyrGTA
5001a	human	tRF-5	chr6-26577332-26577420	chr6.tRNA16-TyrGTA
5001a	human	tRF-5	chr6-26569086-26569176	chr6.tRNA14-TyrGTA
5001a	human	tRF-5	chr14-21151432-21151520	chr14.tRNA5-TyrGTA
5001a	human	tRF-5	chr14-21131444-21131351	chr14.tRNA16-TyrGTA
5001a	human	tRF-5	chr14-21128210-21128117	chr14.tRNA17-TyrGTA
5001a	human	tRF-5	chr14-21125716-21125623	chr14.tRNA18-TyrGTA
5001a	human	tRF-5	chr14-21121351-21121258	chr14.tRNA19-TyrGTA
5001b	human	tRF-5	chr8-67026223-67026311	chr8.tRNA5-TyrGTA
5001b	human	tRF-5	chr8-67025602-67025694	chr8.tRNA4-TyrGTA
5001b	human	tRF-5	chr6-26595102-26595190	chr6.tRNA17-TyrGTA
5001b	human	tRF-5	chr14-21151432-21151520	chr14.tRNA5-TyrGTA
5001b	human	tRF-5	chr14-21131444-21131351	chr14.tRNA16-TyrGTA
5001b	human	tRF-5	chr14-21128210-21128117	chr14.tRNA17-TyrGTA
5001b	human	tRF-5	chr14-21125716-21125623	chr14.tRNA18-TyrGTA
5001b	human	tRF-5	chr14-21121351-21121258	chr14.tRNA19-TyrGTA
5001b	mouse	tRF-5	chr3-90279853-90279782	chr3.tRNA792-MetCAT
5001b	mouse	tRF-5	chr13-22023109-22023038	chr13.tRNA983-MetCAT
				
5032c	human	tRF-5	chr1-161440276-161440205	chr1.tRNA69-AspGTC

*, This data is from tRFdb, which shows tRFID from 1001-1042 belongs to tRF-1; 3001-3033 belongs to tRF-3 and 5001-5032 belongs to tRF-5. And the naming rule is always “chrX.tRNA XXX-Amino acid + anticodon”. The anticodon is made up of three nucleotide bases.

**Table 2:** The type and function of tsRNAs for human

Type	Name	tRF ID in tRF database	Function	Reference
tRF-5	tRNA-Gln CTG	tRF-5021	Inhibit translation	(44)
tRF-5	tRNA-GluCTC	tRF-5030	Inhibit the target mRNA	(3, 43)
tRF-3	tRNA-GlyGCC	tRF-3027	Repress the endogenous Replicating Protein A1	(9)
tRF-1	tRNA-SerTGA	tRF-1001	Patriciate in G2-M transition	(4)
5'-tiRNA	tiRNA-Ala	-	Inhibit translation	(33)
tRF-5	tRNA-Leu/tRF/miR-1280	tRF-5019	Repress colorectal cancer cell proliferation	(5)
5'-tiRNA	Several tsRNAs	-	Associated with hepatitis B-associated HCC	(51)
